# 

**Published:** 1881-09

**Authors:** 


### CONTENTS:

ART. I.—Oral Electricity and the New Departure. By Dr. John J. R. Patrick......193
ART. II.—Proceedings of the Georgia State Dental Society......210
ART. III.—Chemistry and Therapeutics. By W. W. Ford, D. D. S......223
ART. IV.—The Chemical and Physical Effects of Fillings upon Teeth. By Thomas Fletcher, F. C. S......231

### EDITORIAL, ETC.

Hard on Dr. Atkinson......235
Southern Dental Association......235
Crude Blunders......236
Editorial Notice......236

### MONTHLY SUMMARY.

Fooled by Temperature......236
Extraordinary Nephritic Calculus......237
Bromide of Ethyl as an Anæsthetic......238
A Death from Ether......238
Keep Posted......238
Alcohol as a Stimulant or Narcotic......239
Drilling Glass......239
National Dental Association......240
Fatty Tumor of the Tongue......240

